# Case Report: Trazodone as a possible treatment for psychiatric adverse events related to ALK inhibitors

**DOI:** 10.3389/fpsyt.2026.1718767

**Published:** 2026-06-30

**Authors:** Luca Persico, Andrea Miuli, Stefania Chiappini, Alessio Mosca, Michele De Tursi, Mauro Pettorruso, Giovanni Martinotti

**Affiliations:** 1 Department of Neurosciences, Imaging and Clinical Sciences, Università degli Studi G. D’Annunzio, Chieti, Italy; 2 Department of Mental Health, Azienda Sanitaria Locale (ASL) 02 Lanciano-Vasto-Chieti, Chieti, Italy; 3 Psychopharmacology, Drug Misuse and Novel Psychoactive Substances Research Unit, School of Life and Medical Sciences, University of Hertfordshire, Hatfield, United Kingdom; 4 Department of Innovative Technologies in Medicine and Dentistry, University G. D’Annunzio, Chieti-Pescara, Italy; 5 Department of Pharmacy, Pharmacology, Clinical Science, University of Hertfordshire, Hatfield, United Kingdom

**Keywords:** alcohol, lornatinib, metastatic non-small cell lung cancer, psychiatric adverse events, trazodone

## Abstract

**Introduction:**

Lorlatinib is a third-generation tyrosine kinase inhibitor approved for the first-line treatment of ALK-positive metastatic non-small cell lung cancer, frequently associated with psychiatric adverse events.

**Method:**

This study presents the clinical case of a patient who was successfully treated with low-dose trazodone for lorlatinib-induced psychiatric symptoms. In addition, an overview of the existing literature on the management of psychiatric adverse events (AEs) associated with ALK inhibitors is provided.

**Results and conclusions:**

Based on the evidence presented and the preliminary outcomes observed in the reported case, this study aims to outline a management strategy for lorlatinib-related psychiatric complications.

## Introduction

Lorlatinib, a third-generation tyrosine kinase inhibitor (TKI), is indicated, in accordance with international guidelines (1, 2), for the first-line treatment of ALK-positive metastatic non-small cell lung cancer (NSCLC) with high selectivity for ALK/ROS1 genetic aberrations (3). In approximately 60% of patients treated with lorlatinib, neurocognitive and psychiatric adverse events, such as psychotic, cognitive, mood, and speech changes, are observed compared to other ALK inhibitors. Currently, the exact mechanisms underlying the onset of psychiatric effects are unknown; however, it has been hypothesized that ALK plays a role in the process of internalization of the dopamine D2 receptor (D2R) (4). Lorlatinib has a remarkable ability to enter the brain (5), so there is a potential for the occurrence of adverse events in the central nervous system (3, 6) and for psychiatric disorders to develop (7). In this article, we propose the case of a patient diagnosed with ALK-positive lung cancer who developed psychiatric symptoms during treatment with lorlatinib. Moreover, we provide an overview of the current literature on the treatment of psychiatric adverse events related to ALK inhibitors.

## Case description

The patient observed was a 46-year-old white female patient who was diagnosed with metastatic ALK-positive lung cancer in March 2023. No psychiatric history or central nervous system metastases were noted. The patient reported alcohol use to manage anxiety symptoms. She stopped drinking starting psychopharmacological therapy. On 9 October 2024, a psychiatric consultation was requested at the psychiatric outpatient department of “Ss Annunziata” Hospital (Chieti, Italy), due to the onset of lorlatinib 100 mg/day treatment side effects. In fact, after approximately 6 weeks of lorlatinib treatment, the patient showed affect lability, with uncontrollable crying and a tendency to view her life tragically. The patient also experienced episodes of derealization (“my soul was detached from the body”). Over the following weeks, somatic manifestations merged, such as the perception of “blood in the mouth”and difficulty swallowing due to a sensation of a “foreign body in the throat”. The oncologists decided to suspend lorlatinib and requested a psychiatric consultation. At each follow-up visit, the patient presented detailed analyses of hepatic and renal function, blood counts, and ECGs, all of which were within the normal range.

### Diagnostic assessment

During the psychiatric interview at admission to the Department of Mental Health (October 2024), the patient exhibited significant levels of anxiety with a mild mood deflection. Sleep was seriously affected in all phases. Trazodone 75 mg/day (prolonged release formulation) was administered (One-third of a tablet at 8:00 AM, two-thirds of a tablet at 10:00 PM). After she signed an informed consentform, the patient underwent sociodemographic and psychometric scale assessments at baseline (T0) and at different time points (all psychometric results are shown in **Table 1**). According to the CTCAE of the National Cancer Institute v5.0 (8), the patient was grade 2 at T0. A psychometric trait scale (QUICK-SCID) revealed an anxiety trait at T0; it can therefore be assumed that the patient started with a grade 1 before experiencing lorlatinib side effects. During interviews in the following months, the patient showed a progressive improvement in sleep quality (the ISI score decreased from 6 at T1 to 11 at T4) and a significant reduction in anxiety (the HAM-A score decreased from 27 at T0 to 6 at T3). The HAM-A score (9) quickly dropped to 2 points in the second month and increased to 6 points in the third month. The patient experienced a significant, immediate initial benefit following the reduction of lorlatinib and the introduction of trazodone, with a decrease in state anxiety. Once the acute phase was resolved, the subsequent increase in anxiety scores could be explained by physiological personality traits (i.e., trait anxiety), which were identified during initial screening with personality tests. An improvement in quality of life occurred, presumably attributable to the control of anxiety and insomnia (10), thanks to trazodone. No side effects were reported. These aspects would also explain the improvement in mood, as assessed by MADRS (11) and HAM-D levels (All psychometric values are presented in **Table 1**). At T4, a clinically relevant increase in SHAPS (12) scores was observed despite low MADRS levels; this trend could potentially co-occur with chronic treatment-related fatigue or external psychosocial factors, which may have shared a role in influencing the patient’s hedonic capacity. Initial results from the SCL90-R (a self-report tool that measures overall psychological distress and 9 primary symptom dimensions) showed marked clinical issues, particularly for ANX (77), PSDI (68), and PSY (68), all of which were well above the pathological threshold of 60. GSI indices (60) further confirmed widespread distress and intense symptom perception. Follow-up data showed a marked improvement: all values steadily returned to the normal range, settling between 38 and 53, and restoring the overall profile to a state of psychological balance.

**Table 1:** Psychometric scales scoring.

Psychometric Scales		T_0_	T_1_	T_2_	T_3_	T_4_
ISI		19	6	5	4	11
SHAPS		6	0	0	14	42
HAM-A		27	5	2	6	7
HAM-D		21	8	6	5	3
MADRS		16	5	4	4	4
SSI		0	0	0	0	0
SCL-90R	*GSI*	60	41	38	40	41
	*PST*	54	38	35	39	41
	*PSDI*	68	47	38	38	40
	*SOM*	57	39	39	40	44
	*O-C*	63	43	41	41	41
	*I-S*	46	40	40	40	40
	*DEP*	49	39	39	42	44
	*ANX*	77	46	40	44	44
	*HOS*	44	44	44	44	44
	*PHOB*	55	40	40	43	40
	*PAR*	41	41	41	41	41
	*PSY*	68	53	43	46	43

T0: baseline in October 2024; T1: 1 week after baseline; T2: 1 month after baseline; T3: 3 months after baseline; T4: 4 months after baseline; SHAPS (Snaith–Hamilton Pleasure Scale); ISI (Insomnia Severity Index); HAM-A (Hamilton Rating Scale for Anxiety); HAM-D (Hamilton Rating Scale for Depression); MADRS (Montgomery Äsberg Depression Rating Scale); SCL-90 R (Symptom Checklist-90): GSI (Global Severity Index), PST (Positive Symptom Total), PSDI (Positive Symptom Distress Index), SOM (Somatization), O-C (Obsessive-Compulsive), I-S (Interpersonal Sensitivity), DEP (Depression), ANX (Anxiety), HOS (Hostility), PHOB (Phobic Anxiety), PAR (Paranoid Ideation), PSY (Psychoticism); SCL-90-R scoring: Results are interpreted using normalized T-scores, where a T-score ≥ 63 (or a raw score above the 90th percentile) is typically considered the threshold for clinical significance; ISI scoring: 0–7 no clinically significant insomnia; 8–14 mild insomnia; 15–21 moderate clinical insomnia; 22–28 severe clinical insomnia; SHAPS Scoring: 0–2 normal hedonic capacity, ≥ 3 presence of anhedonia; HAM-A Scoring: ≤ 17 mild anxiety, 18–24 moderate severity, ≥ 25 severe anxiety; HAM-D Scoring: Scores between 0–7 normal range; 8–17 mild depression; 18–24 moderate depression; scores ≥ 25 severe depression; MADRS Scoring:: 0–6 normal/clinical remission, 7–19 mild depression, 20–34 moderate depression, ≥ 35 severe depression; SSI Scoring: 0: absence of suicidal ideation, any score above 0 necessitates immediate clinical attention. A score of ≥ 6 is generally recognized as an indicator of significantly elevated risk that requires urgent psychiatric intervention.

### Literature overview

An overview of the current literature was conducted by using three databases (Scopus, PubMed, and Web of Science) on 1 March 2025, using the following strings: “(lorlatinib OR Lorviqua) AND (anxiety OR depression OR psychosis) NOT (review OR animal OR mice)”. After excluding duplicates and articles not related to the focus of the overview, 9 articles written in English were considered in this article (**Table 2**). In addition to these results, data and technical product information from an EMA were added. The current scientific literature on the adverse effects of lorlatinib use identifies a group of neurocognitive adverse events (NAEs) including psychotic alterations (onset at 23 days) and mood changes (median time to onset at 43 days). These psychiatric effects are generally mild, transient, spontaneously reversible, or reversible after a dose reduction of lorlatinib (13–15). The median time for CNS improvement was typically of 12.5 days (range: 2–112 days) (16). Several treatments have been proposed, especially for the treatment of anxiety and depression. In particular, it is advisable to use antidepressants with an anxiolytic profile (since benzodiazepines have a negative effect on cognition and saturation) favoring duloxetine and agomelatine. Caution is advised when using paroxetine, fluoxetine, citalopram, escitalopram, sertraline, fluvoxamine, venlafaxine and mirtazapine. Non-pharmacological treatments include psychotherapy and mindfulness (4). Four grades, in accordance with the National Cancer Institute’s Common Terminology Criteria for Adverse Events (CTCAE), are used to classify the severity of psychiatric side effects (8). Each of these grades indicates a specific lorlatinib dosage adjustment. Grade 1: maintain or reduce the dose of lorlatinib; Grades 2-3: lorlatinib should be discontinued until toxicity is below grade 1, at which point the drug can be reintroduced at a lower dose; Grade 4: lorlatinib should be permanently discontinued. To identify any pre-existing problems, all patients are recommended to undergo mental status and neurological examinations before initiation of lorlatinib therapy.

**Table 2:** Article included.

Authors	Article title	Publication year	DOI
Liu, MA; Xu, E; Shu, CA	A case study on severe psychiatric symptoms induced by lorlatinib	2024	10.1002/pon.6283
John, A; Vick, J; Sarker, S; Middleton, E; Cartwright, E; Manickavasagar, T; McMahon, D; Tokaca, N; Popat, S	Successful Lorlatinib Rechallenge After Severe Drug-Induced Psychosis in ALK-Positive Metastatic NSCLC: A Case Report	2024	10.1016/j.jtocrr.2024.100689
Fallet, V; Rouby, P; Ahle, G; Arrondeau, J; Naltet, C; Duflot-Boukobza, A; De Crozals, F; Lena, H; Cortot, A	Central nervous system disorders on lorlatinib: How to detect and manage in practice?	2022	10.1016/j.bulcan.2022.01.011
Kunimasa, K; Wada, M; Nishino, K	Severe Psychosis Associated with Lorlatinib	2023	10.1016/j.jtho.2023.03.025
Barata, F; Aguiar, C; Marques, TR; Marques, JB; Hespanhol, V	Monitoring and Managing Lorlatinib Adverse Events in the Portuguese Clinical Setting: A Position Paper	2021	10.1007/s40264-021-01083-x
Sisi, M; Fusaroli, M; De Giglio, A; Facchinetti, F; Ardizzoni, A; Raschi, E; Gelsomino, F	Psychiatric Adverse Reactions to Anaplastic Lymphoma Kinase Inhibitors in Non-Small-Cell Lung Cancer: Analysis of Spontaneous Reports Submitted to the FDA Adverse Event Reporting System	2013	10.1007/s11523-021-00865-8
Arriola E.; de Castro J.; García-Campelo R.; Bernárdez B.; Bernabé R.; Bruna J.; Dómine M.; Isla D.; Juan-Vidal Ó.; López-Fernández T.; Nadal E.; Rodríguez-Abreu D.; Vares M.; Asensio Ú.; García L.F.; Felip E.	Expert Consensus on the Management of Adverse Events of Lorlatinib in the Treatment of ALK+ Advanced Non-small Cell Lung Cancer	2024	10.1007/s40261-024-01379-7
Li H.; Wang C.; Guo C.	Post-marketing safety of lorlatinib: a real-world study based on the FDA adverse event reporting system	2024	10.3389/fphar.2024.1385036
Jia K.; Ren S.	Neurocognitive Adverse Events of Lorlatinib: On the Way to Precise Prediction?	2023	10.1016/j.jtho.2022.11.003

## Discussion

The development of ALK TKIs has improved the survival outcomes for patients with advanced ALK-rearranged NSCLC. AEs related to ALK inhibitors are well known; notably, approximately 20% of patients receiving lorlatinib experienced cognitive effects and behavioral alterations in several trials (17).

The patient presented in this article experienced regression of her somatic symptoms due to reduced lorlatinib dosage, although persisting insomnia and affective symptoms such as nervousness, affect lability, and mood deflection. After a clinical interview and psychometric evaluation at T0, trazodone (75mg/day) in prolonged-release formulation was administered to evaluate its efficacy in treating affective symptoms and insomnia (18, 19). Trazodone, in particular, acts primarily as a serotonin receptor antagonist 5-HT2A and also has serotonin reuptake inhibition with antihistamine properties (17, 18). Lorlatinib is a moderate-to-strong inducer of the CYP3A4 cytochrome. Fluvoxamine is a potent inhibitor of CYP3A4 and CYP1A2; its use with lorlatinib is problematic as it can alter the levels of the oncological drug, and vice versa. Other SSRIs, such as paroxetine and fluoxetine, are potent inhibitors of CYP2D6. Although trazodone is metabolized by CYP3A4, the induction caused by lorlatinib might, at most, slightly reduce its plasma levels (requiring a minor dosage adjustment), but it does not create a dangerous metabolic blockade or increase the toxicity of the oncological drug, making trazodone more manageable. The patient reported anxiety and insomnia, and SSRIs can cause, especially in the early stages of treatment, activation, restlessness, and a worsening of insomnia. These effects may vary depending on the dose and formulation used. Regarding the patient mentioned in this case report, it was decided to choose trazodone for her good handling of the drug, thanks to its different formulations that allow for personalization of the treatment, optimizing clinical results and minimizing side effects (18, 19). While trazodone is primarily approved as an antidepressant, according to the FDA, its off-label use for conditions such as insomnia and anxiety is likely its most prevalent application (20, 21).

### Clinical implications

According to Barata et al. (4), we propose the possible management of lorlatinib-induced psychiatric AEs:

#### Before starting lorlatinib

Require a psychiatric assessment to investigate the patientt’s history and administer a mental status examination (the QUICK-SCID was used in this study, but any choice of trait scale based on skills and availability in the clinical setting is fine) in order to identify a pre-existing psychiatric condition. If the initial psychiatric consultation highlighted any pre-existing conditions, it would be appropriate to administer therapy if needed (defining a follow-up) or re-evaluate the patient within several weeks, considering the median timing of the occurrence of AEs (43 days for mood side effects and 23 days for psychotic effects).

#### After starting lorlatinib

*If Psychotic AEs occurred.*

Support medication second-generation antipsychotics [olanzapine is recommended; quetiapine and ziprasidone may have interactions with lorlatinib, while clozapine and risperidone should be used with caution (23)].

Lorlatinib adjustments treatment should be interrupted in the presence of symptoms perceived as moderate to severe (grade 2 or higher) and resumed upon improvement to grade 1.

Monitoring observe and question the patient during follow-up consultations. MRI or CT should be performed every 6 weeks.

*If Mood AEs occurred.*

Support medication treat anxiety and/or depression, favoring duloxetine and agomelatine. Caution is suggested when using paroxetine, fluoxetine, citalopram, escitalopram, sertraline, fluvoxamine, venlafaxine, and mirtazapine. To relieve anxiety symptoms, benzodiazepines should be the second choice, considering the impact that these drugs have on the respiratory system and cognition (24–26). Trazodone, according to our observations, is a valid choice. Non-pharmacological treatments include psychotherapy and mindfulness.

Lorlatinib adjustments treatment should be interrupted in the presence of symptoms perceived as moderate to severe (grade 2 or higher) and resumed upon improvement to grade 1;

Monitoring observe and question the patient during follow-up consultation. MRI or CT should be performed every 6 weeks.

## Conclusions and study limitations

Treatment with lorlatinib is known to cause psychiatric symptoms. This article presented the case of a patient with psychiatric changes related to the use of lorlatinib, who was treated effectively with trazodone. Although a case report cannot determine guidelines, it does provide preliminary information on the possible role of trazodone as a treatment choice for anxiety and sleep disorder onset during lorlatinib treatment. A limitation of this study is the concurrent dose reduction of lorlatinib, which introduces a potential confounding factor; consequently, the adjusted oncological treatment may have also contributed to the overall symptomatic relief. More studies are needed to confirm the hypothesis shown in this work. Giving more treatment options to clinicians may reduce the discontinuation of treatment due to psychiatric AEs, making it possible to improve the quality of life and life expectancy for these specific, fragile patients.

## Data Availability

The raw data supporting the conclusions of this article will be made available by the authors, without undue reservation.
